# Case Report: Specific ABL-Inhibitor Imatinib Is an Effective Targeted Agent as the First Line Therapy to Treat B-Cell Acute Lymphoblastic Leukemia With a Cryptic *NUP214*::*ABL1* Gene Fusion

**DOI:** 10.3389/pore.2022.1610570

**Published:** 2022-09-12

**Authors:** Egle Stukaite-Ruibiene, Rimvydas Norvilas, Vaidas Dirse, Sigita Stankeviciene, Goda Elizabeta Vaitkeviciene

**Affiliations:** ^1^ Faculty of Medicine, Vilnius University, Vilnius, Lithuania; ^2^ Hematology, Oncology and Transfusion Medicine Center, Vilnius University Hospital Santaros Klinikos, Vilnius, Lithuania; ^3^ Department of Experimental, Preventive and Clinical Medicine, State Research Institute Centre for Innovative Medicine, Vilnius, Lithuania; ^4^ Center for Pediatric Oncology and Hematology, Vilnius University Hospital Santaros Klinikos, Vilnius, Lithuania

**Keywords:** case report, targeted therapy, tyrosine kinase inhibitors, acute lymphoblastic leukemia, imatinib, BCR-ABL1-like

## Abstract

Acute lymphoblastic leukemia (ALL) with recurrent genetic lesions, affecting a series of kinase genes, is associated with unfavorable prognosis, however, it could benefit from treatment with tyrosine kinase inhibitors (TKI). *NUP214*::*ABL1* fusion is detected in 6% of T-cell acute lymphoblastic leukemia (T-ALL), and is very rare in B-ALL. We present a case of adolescent with B-ALL and a cryptic *NUP214*::*ABL1* fusion which was initially missed during diagnostic screening and was detected by additional RNA sequencing. Treatment with specific ABL-inhibitor Imatinib was added later in therapy with a good effect. Initial treatment according to conventional chemotherapy was complicated by severe side effects. At the end of Consolidation, the patient was stratified to a high risk group with allogeneic hematopoietic stem cell transplantation because of insufficient response to therapy. At that time, targeted RNA sequencing detected *NUP214*::*ABL1* gene fusion which was previously missed due to a small microduplication in the 9q34 chromosome region. Gene variant analysis revealed no TKI-resistant *ABL1* mutations; therefore, treatment with Imatinib was added to target the *NUP214::ABL1* fusion protein. A negative minimal residual disease was achieved, and treatment was downgraded to intermediate risk protocol. Combining routine genetic assays with next-generation sequencing methods could prevent from missing atypical gene alterations. Identification of rare targetable genetic subtypes is of importance in order to introduce targeted therapy as early as possible that may improve survival and reduce toxicity. Treatment with ABL1 inhibitor imatinib mesylate revealed as a highly effective targeted therapy against the leukemia driving protein kinase.

## Introduction

Acute lymphoblastic leukemia (ALL) is a heterogeneous disease with certain cytogenetic aberrations being long-recognized to be anticipated an unfavourable prognosis. A new *BCR*-*ABL*-like subgroup of tyrosine-kinase driven ALL has been associated with a poor response to chemotherapy, a high relapse risk, and unfavorable long-term outcomes (1). In the 2016 updated World Health Organization classification of myeloid neoplasms and acute leukemia, *BCR*-*ABL1*-like B-ALL was added as a new provisional entity (2). Its gene expression profile is similar to *BCR*::*ABL1,* however, is lacking *BCR*::*ABL1* fusion (3). The presence of Nucleoporin 214-ABL Proto-Oncogene 1 (*NUP214*::*ABL1*) gene fusion is detected in 6% of T-ALL, whereas it is rare in B-ALL (4). A series of genes that activate tyrosine kinase and cytokine receptor signaling are affected in *BCR*-*ABL1*-like ALL, suggesting the potential interest of targeted treatment with tyrosine kinase inhibitors (TKI) (5). However, the therapeutic effect of TKI for the *NUP214*::*ABL1*-positive patients is disputable as clinical experience is limited (6–11). Standard worldwide screening methods for known *ABL1* gene fusions include fluorescence *in situ* hybridization (FISH) analysis and reverse transcription polymerase chain reaction (RT-PCR) (12). Nevertheless, these screening techniques detect a limited number of alterations as *BCR*-*ABL1*-like ALL is known for its highly heterogeneous background (13).

We present a case of pediatric B-ALL with a cryptic *NUP214*::*ABL1* gene fusion which was initially missed during diagnostic screening due to unusual genetic alteration and was identified by the targeted next-generation sequencing (NGS) only. Treatment with a first-generation TKI (imatinib) was added to the chemotherapy with a good effect.

## Case Description

A 15 year-old boy with no previous significant medical history was admitted to our pediatric department in July 2020 for high fever, petechial and hemorrhagic rash, and vomiting. The blood count showed hemoglobin 51 g/L, platelet count 34 × 10^9^/L, and hyperleukocytosis 464.5 10^9^/L. Immunophenotyping confirmed the expression of B-lymphoid markers CD45, CD19, CD10, CD20, CD81, CD22, cCD22, CD24, and cCD79a. Routine genomic screening by a single nucleotide polymorphism array (SNP-A) detected normal male karyotype 46,XY without larger aberrations in size ≥5 Mb. FISH and RT-PCR did not detect any of the following recurrent rearrangements: *BCR*::*ABL1*, *KMT2A*, *EPOR*, *ABL1*, *ABL2*, *RUNX1* (*CSF1R*), *PDGFRB*, *E2A* (*TCF3*), *JAK2*, *ETV6*::*RUNX1*, or *CRLF2*. Cerebrospinal fluid showed three WBC/μl and ∼5.8% of aberrant phenotype B-lymphoid cells, with no leukemic blasts in cytospin. B-ALL, CNS1 was diagnosed.

**FIGURE 1:**
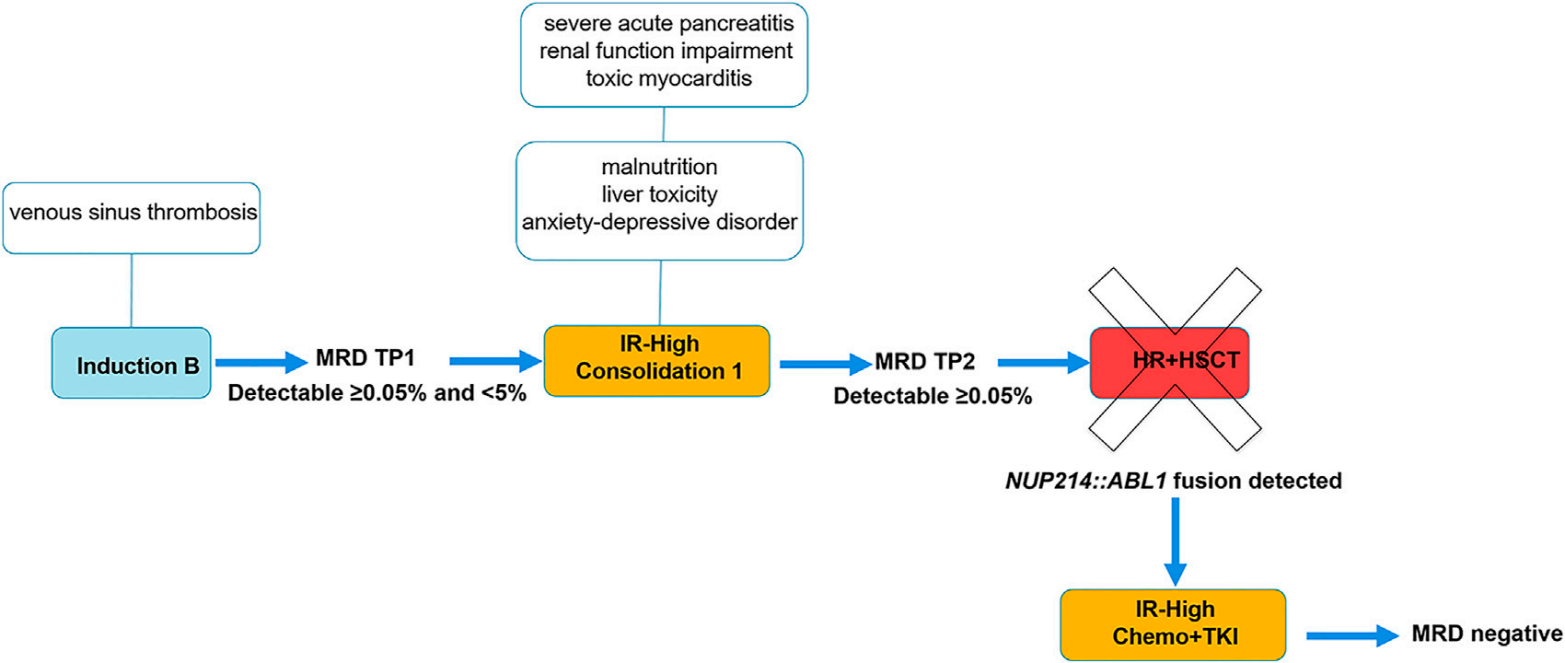
Treatment flowchart and timeline of symptoms. Abbreviations: HR+HSCT, high risk block chemotherapy with hematopoietic stem cell transplantation; IR-High, intermediate-high risk group; MRD, minimal residual disease; TKI, tyrosine kinase inhibitor; TP1, time point 1: end of Induction, day 29; TP2, time point 2: end of Consolidation 1, day 71.

Treatment was conducted according to ALLTogether protocol Induction B with dexamethasone, vincristine, daunorubicin, pegylated-asparaginase (PEG-Asp), and intrathecal methotrexate. At the end of induction, at time point 1 (TP1), minimal residual disease (MRD) in bone marrow showed residual cells of 0.79% by flow cytometry (FC) and 0.03% by IG/TCR quantitative PCR, respectively. Discrepancy in the lab results was interpreted as a subclone of leukemic cells that was not captured by PCR, and the patient was stratified to intermediate-high risk (IR-H) due to a slow response to the therapy as per protocol (Figure 1). Consolidation with dexamethasone, vincristine, 6-mercaptopurine, cyclophosphamide, cytarabine, PEG-Asp, and intrathecal methotrexate was given according to IR-H protocol. Bone marrow evaluation on day 71, time point 2 (TP2) still showed positive MRD: ∼0.18% and 0.07% by FC and IG/TCR quantitative PCR respectively. The patient was stratified into High-Risk (HR) group with allogeneic hematopoietic stem cell transplantation (allo-HSCT) (Figure 1).

Chemotherapy was complicated by multiple side effects. The patient was treated at the intensive care unit twice because of repeated tonic-clonic seizures caused by venous sinus thrombosis in Induction phase and because of severe acute pancreatitis in Consolidation 1. Acute pancreatitis was complicated by multiple organ dysfunction including renal function impairment and toxic myocarditis. Furthermore, the patient suffered from malnutrition, liver toxicity and mixed anxiety-depressive disorder. On day 71 (TP2), targeted RNA sequencing was performed on patient’s RNA sample using TruSight Pan-Cancer sequencing kit as described earlier (14). Sequencing data analysis revealed t(9;9)(q34;q34)/*NUP214*::*ABL1* gene fusion. Exon 33 of the *NUP214* gene and exon 3 of the *ABL1* gene were fused. RT-PCR method was used to confirm *NUP214*::*ABL1* fusion transcript*.* Gene variant analysis showed no TKI-resistant *ABL1* mutations; therefore, treatment with a first-generation TKI imatinib mesylate was added to the conventional chemotherapy. Complete remission (CR) was achieved within a month, and treatment was downgraded to intermediate-risk protocol (Figure 1). At the time of writing the manuscript, the patient is in the first CR 24 months from diagnosis.

The present report was conducted in accordance with the guidelines of the Declaration of Helsinki. Institutional ethical review board permission for a case report was obtained and a written informed consent was received from the patient and his parents.

## Discussion

Cryptic *NUP214*::*ABL1* fusion is a rare genetic entity carrying kinase activating alterations and making the patients candidates for TKI treatment. Although *ABL1* gene rearrangements are most commonly detected in B-ALL, *NUP214*::*ABL1* fusion transcript is mainly described in T-ALL patients (7–9,11,15), whereas in B-ALL its expression is described in individual cases only. In T-ALL, chimeric NUP214::ABL1 protein showed to be sensitive to TKI in preclinical and clinical studies (11), whereas in B-ALL the role of TKI still needs to be established. In pediatric population, approximately 10%–15% of B-ALL cases reveal a *BCR::ABL1*-like profile representing a biologically and clinically challenging group (3). ABL class tyrosine kinase fusion genes are expected to be clonal leukemia drivers and usually respond well to ABL class inhibitors imatinib or dasatinib. Imatinib is generally regarded as the safest of the TKIs, with no long-term irreversible side effects. Although many authors recommend second or third-generation TKIs to override the frequent ATP-binding site mutations (16,17), in our case imatinib showed a very good effect, as there was no evidence of *ABL1* mutations.

To the best of our knowledge, only five B-ALL cases with *NUP214*::*ABL1* fusion caused by intrachromosomal microduplication had been published so far, data are summarized in Table 1. The patients reported were teenagers or young adults, all being >13 years old at the time of diagnosis. Four patients, for whom data was available, had high hyperleukocytosis (WBC >100 × 10^9^/L) at presentation, similarly to our case. In all reported cases, the patients had a poor initial response to therapy and were stratified to very high risk (VHR) chemotherapy (case #4) or allogeneic hematopoietic stem cell transplantation (allo-HSCT) (cases #1 and #5). Two patients received treatment with TKI after allo-HSCT and achieved negative MRD, however, subsequent disease progression in case #4 resulted in lethal outcome. One patient (#5) underwent successful allo-HSCT without additional TKI use.

**TABLE 1:** Cases of B-ALL with a cryptic *NUP214*::*ABL1* fusion.

Case	Age/gender	Karyotype and/or key lesions	WBC, ×10^9^/L	Treatment phase when *NUP214*::*ABL1* detected	Method used for detection	Response to induction treatment	TKI use	Outcome	References
1	26/F	47,XX,inv(9)(p13q34),+10(11)	N/A	End of induction	RNA-seq	Corticosteroid resistance	Dasatinib added to the second Induction cycle and as a single agent started at +35 d. Post allo-HSCT for 23 months, continued at the time of manuscript	CR1	(10)
2	14/M	IKZF1 p.Ser402fs mutation;PAX5deletion;CDKN2A/CDKN2B deletion	220.7	N/A	RNA-seq; confirmed by RT-PCR	N/A	N/A	N/A	(20)
3	16/M	46, XY IKZF1(IK6) and p.Ala79fs mutation	135.6	N/A	RNA-seq; confirmed by RT-PCR	N/A	N/A	N/A	(20)
4	15/F	46,XX	260.0	Disease progression after 1st relapse	High resolution SNP array; confirmed by RT-PCR	Corticosteroid resistance and Induction failure	Dasatinib in combination with chemotherapy at disease progression	CR2 after introduction of dasatinib, however, lethal outcome because of disease progression	(18)
5	13/F	46,XX, t(2;16)(q11.2;q11.2)	480.0	Post allo-HSCT	Targeted RNA; confirmed by RT-PCR	Poor -> allo-HSCT	None	CR1	(19)

allo-HSCT, allogeneic hematopoietic stem cell transplantation; CR1, first complete remission; CR2, second complete remission; N/A, not applicable; RT-PCR, reverse transcription polymerase chain reaction; SNP, single nucleotide polymorphism.

Unlike in previously reported cases, we initiated treatment with a first-generation TKI imatinib mesylate with high efficacy. Two cases (cases #1 and #4) reported a second-generation TKI dasatinib, and a choice of TKI was not specified in the cases #2 and #3. Mechanisms of resistance to imatinib are known to be related to the mutations of ATP-binding site in *BCR*::*ABL1* positive ALL, therefore, dasatinib, nilotinib or ponatinib are preferred as first line therapy (16,17). In our case, gene variant analysis revealed no TKI resistant *ABL1* mutations, which could explain a good effect of Imatinib which was added to the first line of conventional chemotherapy. This subsequently allowed to downgrade the treatment to IR-H risk thus evading allo-HSCT and potentially life-threatening further toxicity.

**FIGURE 2:**
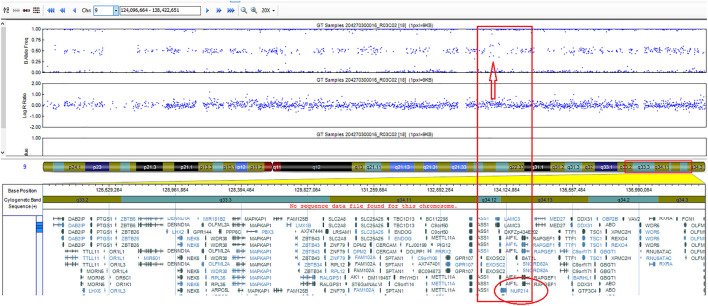
SNP-A karyotyping analysis: 9q34 amplification delimited by *NUP214* and *ABL1* genes.

**FIGURE 3:**
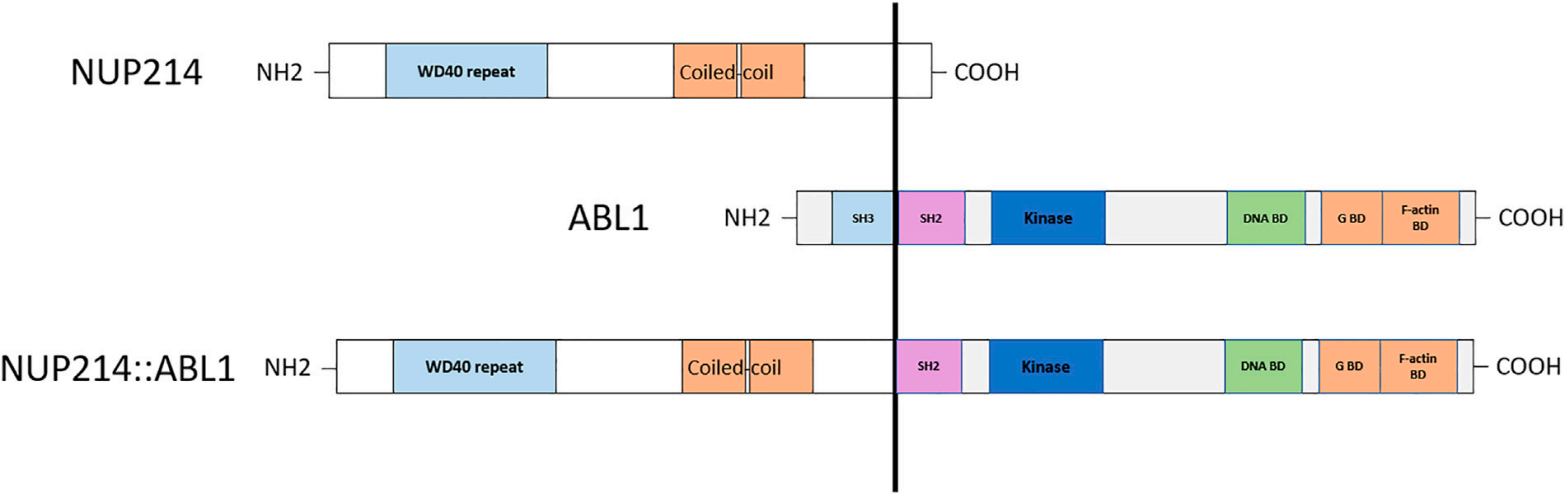
Schematic representation of the chimeric NUP214::ABL1 protein.

Detection of cryptic *ABL1* gene rearrangements by conventional genetic analysis can be a challenge (20, 19). Among the reported cases, *NUP214*::*ABL1* fusion was identified early in treatment, after the first cycle of induction, in one B-ALL case only, using NGS techniques (10). In two cases, the fusion was initially missed by routine diagnostic methods and detected later by SNP-A or targeted RNA sequencing (20, 19) (Table 1). In our case, the *ABL1* gene break was initially missed by FISH array (*ABL1* Break Apart Probe) due to a small 445 Kb microduplication in the 9q34 chromosome region and was detected later by performing targeted RNA sequencing. SNP-A karyotyping missed microduplication which was smaller than 5 Mb in size (Figure 2). *NUP214* and *ABL1* genes are located at the edges of the 9q34 region, therefore, FISH cannot successfully detect *NUP214*::*ABL1* gene fusion due to technical limitations (Figure 3). This particular cryptic fusion mechanism was described in detail by Tsujimoto et al. (20). In our previous retrospective population-based *BCR*::*ABL1*-negative B-other ALL cohort study, we did not detect any ABL-class fusions in pediatric Lithuanian patients (14), making this case to be the only *NUP214*::*ABL1* gene fusion case in Lithuanian childhood B-ALL emphasizing very rare incidence of this aberration. Some authors suggest that all patients with B-ALL should undergo NGS analysis in parallel with conventional genetic screening (13). In our case, adding TKI to the first line treatment enabled us to downgrade the treatment risk group for the patient. However, earlier NGS results detecting targetable genomic alteration would have been beneficial by allowing initiation of targeted therapy and possibly preventing severe drug-induced side effects.

## Conclusion

Identification of rare targetable genetic subtypes is of importance in order to introduce individualized targeted therapy as early as possible to improve survival and reduce toxicity. Combining TKI with chemotherapy for *ABL1* rearranged B-ALL should be considered for the first-line treatment. B-ALL in adolescent patients without detected recurrent cytogenetic or molecular abnormalities (B-others) should be immediately analyzed further by NGS methods to prevent from missing atypical gene alterations.

## Data Availability

The raw data supporting the conclusion of this article will be made available by the authors, without undue reservation.

## Abbreviations

ALL, acute lymphoblastic leukemia; allo-HSCT, allogeneic hematopoietic stem cell transplantation; CR, complete remission; FISH, fluorescence *in situ* hybridization; HR+HSCT, high risk block chemotherapy with hematopoietic stem cell transplantation; ICU, intensive care unit; IR-High, intermediate-high risk group; MRD, minimal residual disease; NGS, next-generation sequencing; PEG-Asp, pegylated-asparaginase; RT-PCR, reverse transcription polymerase chain reaction; SNP-A, single nucleotide polymorphism array; TKI, tyrosine kinase inhibitor; TP1, time point 1: end of Induction, day 29; TP2, time point 2: end of Consolidation 1, day 71; VHR, very high risk chemotherapy.
